# Intention to quit water pipe smoking among Iranian women: a qualitative directed content analysis

**DOI:** 10.1186/s12905-020-00922-w

**Published:** 2020-03-23

**Authors:** Leila Sabzmakan, Fatemeh Eslami, Shirin Shahbazi Sighaldeh, Nkeonye Judith Izuka

**Affiliations:** 1 Department of Health Education & Promotion, Alborz University of Medical Sciences, Karaj, Iran; 2 Department of Reproductive Health, Tehran University of Medical Sciences, Tehran, Iran; 3 Family Physician & Public Health Practitioner, Student at Skills for Change, Toronto, Canada

**Keywords:** Intention, Water-pipe smoking cessation, Theory, Qualitative study

## Abstract

**Background:**

Water-pipe smoking is the most common type of tobacco used among Iranian women. The aim of this study was to explain women’s perceptions of their intention for quitting water-pipe smoking based on the theory of planned behavior.

**Methods:**

The study was a qualitative content analysis which was carried out over 4 months in 2016 in Tehran-Iran. The participants were 26 women ages 18 to 45-years-old who smoked water-pipe and were selected through snowball sampling. The study was performed in hookah cafes, parks, and homes. The data were collected through individual interviews. The interviews were open-ended questions based on the theory of planned behavior. Directed content analysis was used to analyze the data.

**Results:**

Findings showed that women did not intend to quit water-pipe in that time. Main contributing factors influencing not having intention of cessation were positive attitude and false beliefs toward hookah smoking, as well as having peers and family members who smoked water-pipe or approved its use. Although most females realized the obstacles associated with hookah cessation, they believed that quitting water-pipe smoking was up to them and could control more barriers.

**Conclusion:**

Social pressure, positive attitude and false beliefs towards hookah smoking, as well as external and internal obstacles diminished women ‘s intention for cessation. Therefore, it is recommended to apply the theory of planned behavior into behavior change interventions in order to increase the intention to quit water-pipe smoking.

## Background

One type of tobacco use is water-pipe (WP) smoking and it is a centuries-old method of tobacco use [1]. In Middle East, WP has been traditionally used for centuries and in the last 20 years its use has significantly increased [2]. Currently, all across the world, 3 to 3.5 million people use WP annually and this figure is expected to reach 10 million in the year 2030 [1]. School students, university students, and reproductive age women are more WP users compared with other age groups [3, 4].

The prevalence of WP smoking was reported to be as high as 41% among women and it is gaining popularity unlike cigarette smoking [4]. In Middle Eastern countries, WP smoking was generally socially more acceptable than cigarette smoking, and it is the most worrisome aspect particularly among females [4]. Therefore, quitting WP smoking due to common social acceptability may be assumed to be much more difficult than quitting cigarette smoking [5]. The national Stepwise approach to risk factor Surveillance (STEPS) surveys between 2006 and 2009 among Iranian population reported that the prevalence of WP smoking among men ranged from 1.7 to 10.9% and among women it ranged from 0 to 16.8% [6].

A recent systematic review and meta-analysis study reported that WP smoking was significantly associated with adverse consequences such as respiratory diseases (chronic obstructive pulmonary disease, bronchitis and wheeze due to exposure to passive WP smoking), lung cancer, oral cancer, low birth weight, metabolic syndrome, mental health, and cardiovascular disease [7]. In spite of negative impacts of WP smoking, there is a misconception that it is a less harmful form of cigarette smoking and a safer replacement to cigarette [3].

**Fig. 1:**
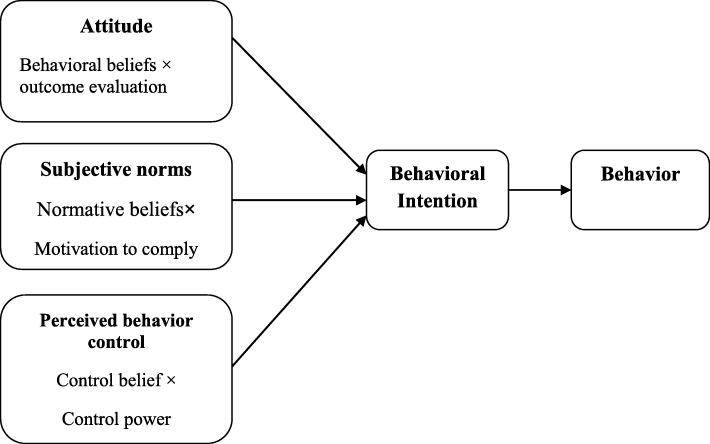
The Theory of Planned Behavior

One of reasons for tobacco smoking is psychosocial aspect, so theory-based interventions were significantly effective on self-efficacy, motivation, beliefs, and awareness of tobacco consumers for cessation [8]. The theory of planned behavior (TPB) is one of theories that is applied to plan smoking cessation. According to the TPB, attitude, subjective norm, and perceived control behavior are primary determinants of a behavioral intention. Attitude is stemmed from beliefs about the likely outcomes of the behavior and the evaluations of these outcomes (behavioral beliefs), subjective norm is originated from beliefs about the normative expectations of others and motivation to comply with these expectations (normative beliefs), and perceived control behavior is guided by beliefs about the presence of the factors that may facilitate or impede performance of the behavior and the perceived power of these factors (control beliefs) [9]. Conceptual of the theory is shown in Fig. 1.

Several studies have identified various reasons for WP smoking such as lack of enforcement of laws prohibiting public consumption [10], considering WP smoking less harmful than cigarettes [5, 11, 12],WP smoking by family and friends [13], failure to resist temptation of WP smoking [14, 15], having entertainment and spending leisure time [12, 16, 17], relaxation and pleasure [5, 12], and social acceptance, easy availability, attractive designs, and flavored aromatic tobacco [12, 18, 19]. Totally, most researches in Iran have focused on the prevalence of WP smoking and its determinants [3, 5, 6, 15, 18, 20]. There are few literatures that examine the intention of smokers for quitting WP smoking [1]. In addition, no theory-based qualitative research to date- to our knowledge- has been published that examined the intention to quit WP smoking among users. The qualitative approach allows researchers to enter into the inner layers of people’s experiences to reach their perception formation in their cultural context [21]. Therefore, the purpose of this study was to explain women’s perceptions of their intention for quitting WP smoking based on the TPB.

## Methods

### Design

This research was a qualitative study with directed content analysis approach. According to the manual of Ajzen to develop the questionnaire of the TPB, first, a qualitative study must be conducted to elicit behavioral beliefs, normative beliefs, and control beliefs [22]. The goal of directed content analysis is to validate or extend a conceptual theoretical framework or theory [23]. Existing theory focuses on the research questions and helps researchers to identify key concepts as initial coding categories. This approach was introduced in 2005 by Shannon and Hsieh [23].

### Data collection

The data were collected via in-depth semi-structured interviews from June until September 2016. Participants were selected using purposive snowball sampling. The interviews were open-ended questions based on the TPB. During the interview and according to the responses, probing questions were also asked. The interview guide was presented in an appendix. The interviews continued until data saturation, such that, at the end of the last two interviews, no new codes were obtained. The interviews were recorded, transcribed verbatim, reviewed, coded and immediately analyzed. In order to capture a deep understanding of participants ‘perceptions, the interviews were read carefully several times.

### Setting and participants

In this study, face-to-face interviews were carried out with 26 women who were WP consumer. The inclusion criteria were ages 18 to 45 years old, women who have lived in Tehran and being current WP smoker (smoked WP at least once in the past month), and willing to participate in the study. The exclusion criteria were suffering from a specific disease, being pregnant, and using of drugs in the past year. Interviews were carried out in places like hookah cafes, parks, and homes and each interview lasted between 30 and 40 min.

### Data analysis

In directed content analysis, coding is done based on the research questions. The important statements were underlined to identify the initial codes or meaning units that exist in the interview text. In the next phase, these similar meaning units (codes) were placed initially into 6 predetermined subcategories of the TPB which include behavioral beliefs, outcome evaluation, normative beliefs, motivation to comply, control beliefs, and control power and into its four main categories which consist intention, attitude, subjective norms, and perceived behavior control. Any text that could not be categorized with the initial coding scheme would be given a new code [23–25]. An example of categories and subcategories of the theory is shown in Table 1.

**Table 1:** Examples of coding and placing them in the categories and subcategories of TPB

Quotes of women who smoked water pipe	Code	Category	Sub-category
“At the moment, I have no intention to quit WP, but I think about reducing”	Not having intention for quitting WP	Intention	
“Quitting WP is useful for my health, it is harmful for lung, heart, teeth and pregnancy”	Quitting WP is in favor of the person	Attitude	
“Quitting WP can lead to having beautiful skin, healthy lung and heart, and teeth”	Advantages/Disadvantages of quitting WP smoking		Behavioral belief
“Having beautiful skin, healthy heart, lung, and teeth are important for me”	Positive or negative evaluation of quitting WP smoking		Evaluation of results
My mom encourages me to quit WP, and she says it is not acceptable for a girl to smoke WP.”	Feeling of social pressure for quitting WP smoking	Subjective Norm	
My friends and family around me smoke WP and none of them want to quit it.”	Individuals who would approve or disapprove quitting WP smoking		Normative beliefs
“I don’t like to quit WP until I really want to do it. Even if my family and friends encourage me to stop it, I may not follow their advices.”	Following/Not following the devices of significant others for quitting WP smoking		Motivation to comply
“I have a great ability to quit WP. If I plan, I will certainly quit it, but it is a difficult task”	People’s confidence that they are capable of quitting WP smoking	Perceived behavioral control	
“I am unable to stop WP because of some barriers such as; it is a fun accessible. Its use is not addictive, and so on.)	Factors that enable person or make it difficult to quite WP smoking		Control belief
“Smoking WP as a fun, not being addictive, social-psychological problems, and having easy access to WP are obstacles that I am unable to quit WP smoking”	Feeling or not feeling in control of quitting WP smoking		Control power

### Ethical considering

Ethical approval was obtained from the Ethics Committee of Alborz University of Medical Sciences (Abzums.Rec.1395.5). All women who participated in the study were informed about the objectives of the research and informed written consent was obtained from all of them. To comply with ethical principles, participants were assured about the confidentiality of their information which is solely used for research purposes.

### Considering of rigor

Prolonged engagement from June until the end of September 2016 provided an opportunity to collect the data. To make sure that the analysis reveals females’ perceptions, member checking was performed during data collection. To confirm dependability and conformability of the data, the initial codes were placed into the subcategories and categories of the TPB, and four experts in the field of health education checked its accuracy. First and second authors of this article, who were expert in health education and familiar with the TPB, investigated external check of the data. Peer checked was also carried out by two experts in health education who had previous experience with the TPB. Maximum variation of sampling also confirmed the conformability and credibility of the data.

## Results

In this study, the average age of females was 36.5 ± 7.08 and the mean years of WP smoking among them was 10.7 years (SD = 3.26). Other demographic characteristics of the interviewees have been shown in Table 2. According to the questions of research, data analysis was resulted in 153 primary codes, which after initial review, first, 101 codes into 6 sub-categories and then into 4 categories of the TPB were placed.

**Table 2:** Demographic characteristics of the participants

Variable	Number	Percent
Marital status		
Single	11	42.3
Married	8	30.77
Windowed	2	7.7
Divorced	5	19.23
Education		
Diploma	8	30.77
Associate	5	19.23
Bachelor and Higher	13	0.50
Occupation		
Housewife	5	19.23
Unemployment	11	42.3
Staff	10	38.46

### Intention to quite WP smoking

Although almost all participants were aware about adverse effects of WP smoking, they did not intend to quit it in that time period. Most of them only wanted to reduce the frequency of it. However, some females expected of themselves, in the future, due to reasons such as marriage or pregnancy would have stopped WP smoking.

*“Currently, I have no intention to quit WP smoking. However, I am going to reduce it, and may slowly stop it. I think if I marry or become pregnant, I'm going to quit it” (WP smoking for 12 years).*

### Attitude towards quitting WP

Most participants considered the use of WP enjoyable and pleasant. Although most of them believed that would benefit from quitting WP smoking, they felt its cessation is unpleasant. The females did not have positive attitude toward quitting WP. Many participants believed that WP is less harmful than cigarette to health because they believed that the passing of the smoke through water deletes toxins. They were also willing to smoke flavored WP and believed that it was not harmful and addictive.

*“In my opinion, quitting WP smoking may be beneficial, but I don’t really agree with that WP smoking is addictive. So, I think I can quit it anytime that I want. To be honest, I enjoy smoking WP. It gives me pleasant feeling” (WP smoking for 10 years).*

*“I am not thinking about quitting WP smoking right now since I do not feel any risks. I will smoke WP as far as I want unless I feel it hurts me or feel my health is at risk. I don’t think it is bad thing” (WP smoking for 10 years).*

The category of attitude is formed by sub-categories of behavioral beliefs and outcome evaluation. Therefore, women’s attitudes toward quitting WP smoking were categorized in these two sub-categories as follow.

### Behavioral beliefs

On the one hand, most women believed that WP smoking cessation was beneficial and useful to their well-being. For example, they stated the advantages of quitting hookah smoking such as improvement their health, making their skin look better, keeping their respiratory system healthy, making their teeth whiter and healthier, causing them to be more accepted by others, improving heart health, making them a role model for others, and having positive effect on fertility. On the other hand, they claimed that WP smoking relaxed, attracted, entertained, and got rid them of stress and obsessive thoughts, and it was considered a good opportunity for gathering with their friend and family who were hookah smoker. However, few women believed that WP smoking could bring about cancer.

*“Quitting WP smoking increases health. If I stop it, my teeth will not change color to yellow; my skin will become look better. One of the advantages is when walking or exercising I will not suffer from shortness of breath. It is even good to my heart. From a social viewpoint, smoking WP is not acceptable” (WP smoking for 15 years).*

*“Quitting WP is even good for my child’s health and makes me look better in front of people. It is also good for people around me since they will not be exposed to secondhand smoking. If I quit WP, I can be a good role model for others” (WP smoking for 14 years)".*

### Outcomes evaluation

From most participants’ viewpoint, the outcomes associated with WP smoking cessation were evaluated positive. For example, they believed quitting WP smoking had positive consequences such as improving health and bringing about social acceptability. However, most women felt that quitting WP smoking was associated with lack of pleasure, calm, as well as losing gathering with family members and peers who smoked WP. Therefore, these disadvantages were judged negative by most hookah users.

*“Being healthy and having healthy lungs, beautiful skin and teeth and health fertility are important to me. But, the most important disadvantage of cessation is losing gathering with peers and family when smoking WP. It is also important to me that I would lose the pleasure that I experience when smoking WP because I have no problem right now, I do not want to quit it” (WP smoking for 4 years).*

*“Health is important to me. The only downside of stopping WP is that I loss fun and company with my friends. Also, when people ask me to smoke WP with them, if I do not follow them, I may miss them or is mocked by them” (WP smoking for 5 years).*

### Subjective norms

Although some participants stated that their parents or peers who were not hookah smoker encouraged them to quit WP smoking, the majority of WP users claimed that their family members (parents, sibilants, and spouse) and peers smoked WP and did not ask them to quit hookah smoking. Therefore, most females under social pressure are encouraged to smoke WP and not be motivated for cessation.

*“My mother hates WP smoking and advises me to quit it because she believes that it is harmful for me. But some of my sisters and brothers and a lot of people around me smoke WP” (WP smoking for 14 years)".*

*“My father does not like me to smoke WP, and I try not to smoke WP in front of my father. Also, one of my sisters disagrees with my WP smoking. However, my husband, another sister and some of my relatives consume WP” (WP smoking for 15 years)*

The category of subjective norm consists of sub-categories of normative beliefs and motivation to comply. Therefore, perceived social pressure was categorized in these two sub-categories as follow.

### Normative beliefs

Most participants stated that among family members, peers, and relatives, their family members, who did not use WP, were more likely to encourage them for quitting WP smoking. However, many women, who their family members and peers were WP smoker, stated that the behavior of WP smoking was approved by them, so the participants did not have a role model to quit WP. In addition, most participants stated that their smoker peers played a vital role in continuing WP smoking.

*“Around me, a lot of people smoke WP; my sisters, many of my friends, and some relatives especially when we get together, and as far as I know, none of them intends to quit WP smoking” (WP smoking for 7 years).*

### Motivation to comply

Although some participants said that family members’ advice especially their parents was important to them, they were not following their advice. Almost all females were willing to smoke WP and stated a little bit motivation to comply with significant others ‘expectations. Only a few of them tried to smoke WP outside home at the request of family members. In addition, they believed that even if people around them, who smoked WP, stopped WP smoking, it would not influence their behavior. As a result, most participants were not paying great attention to significant others ‘expectation and maintained that would quit WP smoking only when they wanted.

*“I don’t stop WP smoking unless I want, even if my family or my husband asks me to do. I should myself realize that WP smoking is not good for my health and decide quitting it. Others expectation for cessation is not useful to me and I do not care them” (WP smoking for 3 years).*

*“I try to not smoke anywhere because of people’s gossip. For instance, I do not smoke WP in front of my relatives or parents. I do not follow anyone as role model to quit unless I decide that I want to quit” (WP smoking for 15 years).*

### Perceived behavioral control

A lot of women felt a high self-efficacy for cessation and though they were able to quit the use of WP anytime wanted. Indeed, they believed that quitting WP smoking was up to them. In addition, most women believed that WP smoking was not addictive, so its cessation would not be difficult and did not think quitting WP was beyond their control. However, some women felt several factors hindered hookah smoking cessation. For example, when the females were tempted, they could not resist WP smoking.

*“I see a lot of ability in myself to quit WP smoking and whenever I want, I will quit it without difficulty. But I might sometimes get tempted again” (WP smoking for 6 years).*

*“I have high ability to quit hookah because it is just fun for me and an excuse to get together with friends, but I am able to quit it if I want” (WP smoking for 10 years).*

The category of perceived behavioral control comprises sub-categories of control beliefs and control power. Therefore, women ‘perceptions about how to deal with the obstacles and whether quitting WP smoking is easy or difficult were categorized in the two sub-categories as the following.

### Control beliefs

Almost all participants believed that their willingness for cessation was more important than other factors. However, they pointed out that some obstacles for cessation such as lack of laws that prohibited WP smoking in public places, lack of awareness and education about adverse effects of WP smoking, having peers or family who smoked WP or approved its use, the existence of hookah cafes and restaurants, having easy access to WP in anywhere, lack of beneficial recreational activities, more prevalent its use in today’s society especially among women, psychological dependency, socio-psychological problems such as unemployment, stress, and nervousness. Also, having false beliefs such as WP was not addictive and harmful were mentioned as other barriers.

“*There are a vast number of hookah cafes, and one can have easy access to WP at their home. I smoke WP when I am upset or nervous. I remember when I heart break; I used to smoke WP every day. All these reasons discouraged me from quitting WP smoking” (WP smoking for 4 years).*

*“Divorce, unemployment, and financial problems cause to go towards WP smoking. I am unable to find solutions for my problems. So, the use of WP is the easiest way to calm myself down” (WP smoking for 11 years).*

### Control power

Participants believed that were able to control more obstacles of quitting WP smoking. However, they pointed out that some barriers were related to organization and community levels and the government and policymakers were responsible for solution them. Some of these barriers were lack of beneficial recreational activities in the community, the existence of hookah cafes and restaurants, having easy access to WP, socio-psychological problems and lack of efficient educational programs.

*“We women cannot control some problems. The government is responsible. For example, I and many my friends who smoke WP are unemployed and staying at home. Some my friends are divorced. Some of them have a lot of conflicts with their husband because of financial problems or cheating in their relationship. In addition, people do not have access to beneficial and inexpensive recreational activities. As you know, hookah smoking is an accessible and low-cost fun, so it is considered as a good replacement in these situations. It gathers people and helps to forget their problems” (WP smoking for 23 years).*

## Discussion

This study was carried out to explain women’ perceptions of their intention for quitting WP smoking based on the TPB. Findings indicated that almost all participants did not intend to stop WP smoking in that time period. The most important factors influencing having no intention for cessation were positive attitude and false beliefs toward hookah smoking, social pressure, and low motivation to comply with significant others ‘expectations. Although most women felt some internal and external obstacles for cessation, they estimated a high self-efficacy to overcome the barriers.

The construct of intention is central to the TPB. Intentions capture the motivational factors that influence a behavior [9]. In the present study, there was no intention for quitting WP smoking among women. Indeed, they only wanted to reduce the frequency of it. Consistent with these findings, the studies by Jawad et al. [26], Wong [27], Abughosh et al. [28], Athamneh et al. [29] and Ward et al. [1] confirmed lack of intention for smoking cessation .

Attitude toward behavior is a person’s overall evaluation of the behavior [30]. It assumed two components lead to the formation of an attitude: beliefs about consequences of the behavior (behavioral belief) and positive or negative judgments about each of these features of the behavior (outcome evaluations) [30]. In this study, most women did not have a positive attitude towards cessation because they believed that WP smoking was enjoyable and pleasant. This finding is in line with the studies by Baheiraei et al. [31], Sabzmakan et al. [32], Sabahy [33] and Smith-Simone et al. [34]. However, in the study by Athamneh et al. [35] WP smoker had positive attitude toward quitting WP smoking. This difference can be attributed to the type of study and target population. In a systematic review study [5], the willingness to quit WP varied across settings. In addition, most participants pointed out that quitting WP smoking increased their health status which was confirmed by the studies of Akl EA [5] and Athamneh et al. [35]. Furthermore, most females believed that WP smoking not only was not addictive and hazardous but also it was socially acceptable, alleviated their stress, and gathered them with their peers and families. The studies by Ward et al. [1] and Akl EA [5] confirmed these findings. This means that females in favor of WP smoking stated it as an enjoyable entertainment leisure activity, no matter the drawbacks. Consequently, hookah smoker females should aware its drawbacks outweigh the benefits.

Subjective norms are a person’s judge of the social pressure to perform or not perform the target behavior. Subjective norms are assumed to have two components which work together; beliefs about how important people would like them to behave (normative beliefs) and motivation to comply with significant others’ expectations (motivation to comply) [30]. In current study, most women believed that their family members and peers smoked WP or approved its use. Ward et al. [1], Akl EA [5], Baheiraei et al. [13], and Momenabadi et al. [20] stated that the influence of social pressure was a significant factor for starting or quitting smoking. Moreover, in Iranian culture, WP smoking is considered as a favorable method by some people to gather with their family members, relatives, and friends. This finding was confirmed by Baheiraei et al. [13] and Momenabadi et al. [20]. In current study, only families who did not smoke WP expected or encouraged participants to quite WP smoking. However, most females said that they did not follow their significant people’s expectation. The study by Athamneh et al. [29] showed that high motivation to comply was significantly linked with the intention to quite WP smoking. This difference can be assigned to the type of study. As was mentioned in current qualitative study, we examined women ‘perceptions about motivation to comply for cessation whereas in Athamneh’s study this construct was evaluated using a questionnaire. Therefore, social pressure plays a vital role in quitting WP smoking. It seems to enable women avoid and resist negative social pressure.

Perceived behavioral control is the extent to which a person feels ability to do the behavior [30]. It has two aspects; person ‘s self-efficacy and their beliefs about controllability of the behavior. It is determined by control beliefs and power of both external and internal factors to inhibit or facilitate the performance of the behavior [30]. In this study, most participants estimated a high self-efficacy for quitting WP smoking. Indeed, they claimed that quitting WP smoking was up to them. They believed that could overcome more obstacles and quit it anytime wanted. Findings of the studies by Ward et al. [1], Akl EA [5], Athamneh et al. [35], and Momenabadi et al. [20] confirmed our results. However, in the study by Almerie et al. [36], most participants believed that WP smoking cessation was difficult. According to Athamneh et al. [35], perceived behavioral control has been proven influential to modify non volitional behaviors. Moreover, some researchers [37–39] reported that perceived behavioral control as strong predictor of the intention for smoking cessation. In fact, most theory-based quantitative studies indicated perceived behavioral control as a significant factor for not quitting smoking. However, in current qualitative study, perceived behavioral control was not a contributing main factor for cessation. This difference can be attributed to the type of research. Furthermore, many females because of having wrong beliefs such as not being addictive and harmful WP smoking were unable to stop it. Moreover, the existence of some environmental factors such as absence of laws that prohibit WP smoking in public places, lack of beneficial recreational activities, the existence of hookah cafes and restaurants, having easy access to hookah anywhere, socio-psychological problems, lack of campaign for public education were realized as the drawbacks that diminished the ability of the females for cessation. These findings are consistent with results of studies which were conducted by Momenabadi et al. [20], Akl EA [5], and Ward et al. [1]. As a result, it is necessary for women to be enabled how to deal with obstacles of quitting WP smoking.

### Limitations of this study

This study was a qualitative theory-driven study and its findings provided a deep understanding of the factors associated with women’s intention for quitting WP smoking. This helped to develop a questionnaire based on the TPB that could not be developed through quantitative study. The generalizability of the findings may be limited because interviews were conducted with females who were introduced to researchers and those who agreed to participate in the interview. On the other hand, these participants may not be representative of all females who smoked hookah because some participants avoided expressing their real experiences. Furthermore, all participants of the study were from Tehran and their experiences may not be generalized to women from other cities of Iran. Hence, this limitation influences the applicability of the findings. The findings of current study could support the subcategories and categories of the TPB. Moreover, the data were analyzed appropriately and results were corroborated by using of multiple reviewers especially correspondence to the researchers who were familiar with the TPB to ensure that participants’ viewpoints were adequately interpreted.

## Conclusion

Overall, the trend towards excessive usage of WP among women and its negative impact on health is a concern. Social pressure, positive attitude and false beliefs towards WP smoking as well as external and internal obstacles diminished women ‘s intention to quit WP smoking. Therefore, it is suggested that behavior change interventions are designed on the basis of the TPB to modify factors associated with the intention for quitting water-pipe smoking.

## Data Availability

The datasets generated during the study are not publicly available to protect the participants’ anonymity, but they are available from the corresponding author on reasonable request.

## Abbreviations

WP: Water-pipe
TPB: Theory of planned behavior
STEPS: Stepwise approach to risk factor Surveillance
